# Newly Developed Polyglycolic Acid Reinforcement Unified with Sodium Alginate to Prevent Adhesion

**DOI:** 10.1155/2018/4515949

**Published:** 2018-04-03

**Authors:** Shinichiro Morita, Toshitaka Takagi, Rie Abe, Hiroyuki Tsujimoto, Yuki Ozamoto, Hiroko Torii, Akeo Hagiwara

**Affiliations:** ^1^Faculty of Life and Medical Sciences, Department of Medical Life System, Doshisha University, 1-3 Tatara Miyakodani, Kyotanabe, Kyoto 610-0394, Japan; ^2^Fushimi Okamoto Hospital, 9-50 Kyomachi, Hushimi-Ku, Kyoto 612-8083, Japan; ^3^Kusatsu General Hospital, 1660 Yabashi, Kusatsu, Shiga 525-8585, Japan

## Abstract

Polyglycolic acid (PGA) mesh fabric is widely used for reinforcing injured tissues during surgeries. However, PGA induces chronic inflammation and adhesion. The purpose of this study is to develop PGA reinforcement “without PGA-induced adhesion.” We developed a reinforcement fabric unified with PGA mesh and alginate foam. The antiadhesive effects of sodium alginate foam and calcium alginate foam were evaluated in rats. Sodium alginate foam unified with PGA mesh fabric exhibited strong effects that limit the extent and severity of adhesion, whereas calcium alginate foam unified with PGA mesh was less effective in preventing adhesion. In the sodium alginate group, fibroblasts and collagen fibers around implanted sites were sparse and the material degraded rapidly by macrophage ingestion. Fibroblasts and collagen fibers play a major role in adhesion formation and their excessive proliferation results in postoperative adhesion. Thus, inhibiting their increase is the key in preventing PGA-induced adhesion. The reinforcement that is composed of PGA mesh unified with sodium alginate foam strongly inhibited PGA-induced adhesion and showed excellent handling during surgery and could be easily applied with a one-step procedure.

## 1. Introduction

The use of polyglycolic acid (PGA) mesh in various types of surgery is increasing because of its superior reinforcing effects. The application of reinforcement for automatic suturing device is expanding and increasing. PGA mesh fabric is widely used for reinforcing injured tissues during surgeries [1, 2]. However, PGA induces chronic inflammation and adhesion [3–6]. Adhesion could result in bowel obstruction, infertility, chronic abdominal pain, and difficulties in subsequent surgeries [7–10] as well as prolonged hospitalization and hospital readmissions [11], which in turn could raise hospital costs [12].

Moreover, PGA mesh fabric turns into glycolic acid in vivo, which induces adhesion around the site where PGA mesh is placed (PGA-induced adhesion) [6]. Currently, preventing PGA-induced adhesion is an extremely important concern. Thus, the purpose of this study is to develop a PGA reinforcement “without PGA-induced adhesion.” It has been proposed that alginate has an antiadhesive effect [4, 13, 14]. Our previous study showed that alginate salt (gel or solution) effectively prevents PGA-induced adhesion [4]. However, alginate gel or solution is difficult to handle and has poor retentivity. In this study, we developed a reinforcement fabric unified with PGA mesh and alginate foam to prevent PGA-induced adhesion and to improve its usability during surgery. The ease in use of the newly developed reinforcement and its ability to prevent adhesion were evaluated.

## 2. Materials and Methods

### 2.1. Preparation of Materials

**Figure 1:**
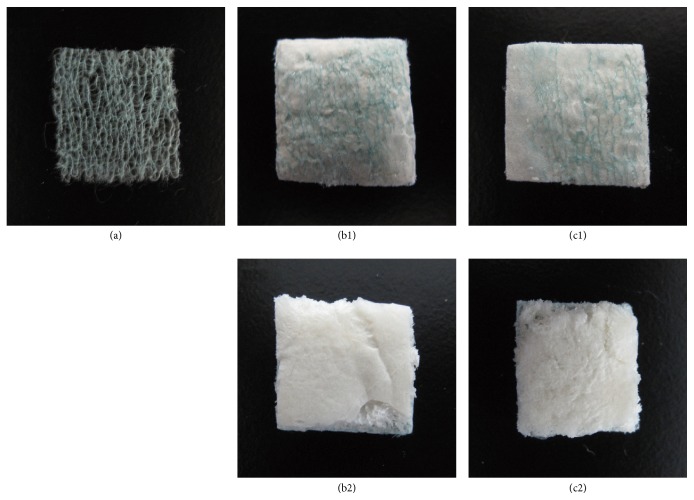
(a) PGA mesh, (b1, b2) PGA mesh with sodium alginate foam (Na-alg), and (c1, c2) PGA mesh with calcium alginate foam.

(I) PGA mesh (NEOVEIL®, Gunze, Kyoto, Japan) was used as the control fabric (Figure 1(a)) and PGA mesh-based reinforcement unified with each of the two kinds of alginate was prepared for this study.

(II) Sodium alginate foam (PGA/Na-alg) was prepared as follows: Square PGA mesh (10 cm) was placed at the bottom of a silicon-coated square container (10 × 10 × 1 cm). Na-alg powder (3.55 g) (Alto®, Kaigen, Osaka, Japan) with molecular weight ranging between 32,000 and 250,000, which is commercially available as a hemostatic agent, was dissolved in 96.45 g of saline. All the Na-alg solution was poured into the container and was frozen at −80°C for 30 min. The frozen Na-alg with PGA mesh was freeze-dried for 24 h, which subsequently turned into a foam. Figures 1(b1) and 1(b2) show the PGA mesh unified with Na-alg foam.

(III) Calcium alginate foam (PGA/Ca-alg) was prepared as follows: Square PGA mesh (10 cm) was placed at the bottom of a 10 × 10 × 1 cm container. Calcium gluconate solution (8.5 wt%; 1 ml) (Calcicol®, Nichiiko, Toyama, Japan) containing 7.85 mg of calcium was added to 100 g of Na-alg solution (3.55 wt%). The alginate partially (<5%) cross-linked by Ca^2+^ was poured into the container, and the solution was frozen. The frozen Ca-alg with PGA mesh was freeze-dried. Figures 1(c1) and 1(c2) show the PGA mesh unified with Ca-alg foam.

All reinforcements were cut into 15 mm square sheets and were sterilized with ethylene oxide for 22 h. Thereafter, the ethylene oxide gas was removed under decompression condition for a week.

### 2.2. Animal Protocol

Fifty-four 9-10-week-old female Wistar/ST rats weighing 200 g (SHIMIZU Laboratory Supplies Co., Kyoto, Japan) were used in this study. During the experimental period, all rats were housed separately and maintained under standard specific pathogen-free (SPF) conditions (light-dark cycle of 12:12 h, temperature of 20.1–23.5°C, and humidity of 37–65%). Standard laboratory rodent chow and water were available ad libitum. The rats were housed in the laboratory for 2 weeks before the experiments. On the day of the experiment, the rats' health condition was assessed. Thereafter, they were randomly assigned into nine groups (6 rats/group).

All rats received isoflurane inhalation anesthesia (Escain®, Mairan Pharmaceutical, Osaka, Japan), and after the experiments, the lethal dose of sodium pentobarbital (75 mg/kg of body weight) (Somnopentyl®, Kyoritsu Seiyaku, Tokyo, Japan) was administered into the abdominal cavities. All surgical procedures and anesthesia administration were performed in accordance with the Animal Care Guidelines of Doshisha University.

### 2.3. Experimental Design

The rats were divided randomly into three groups: Na-alg group, Ca-alg group, and PGA alone group. The ability of the newly developed reinforcement to prevent PGA-induced adhesion was evaluated macroscopically and microscopically.

### 2.4. Surgical Techniques

**Figure 2:**
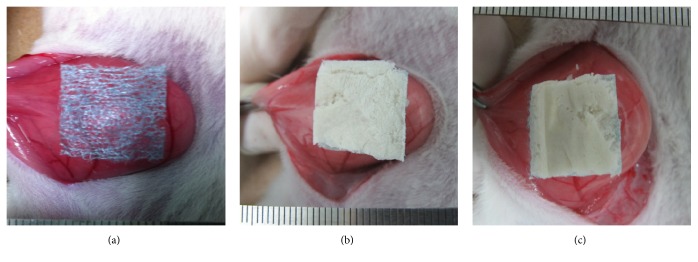
Each material was fixed onto the peritoneum of the right lateral abdominal wall. (a) PGA mesh, (b) Na-alg, and (c) Ca-alg.

The rats were fixed in the dorsal position under general anesthesia. A 4 cm midline incision was made for laparotomy. The material (mesh) was put on the peritoneum of the right lateral abdominal wall, and 0.35 ml of saline covered the entire fabric using 1 ml TERUMO syringe (TERUMO, Tokyo, Japan). Each material was fixed onto the peritoneum with 7-0 polyvinylidene fluoride monofilament sutures (Asflex®, Kono Seisakusho Co., Chiba, Japan) at the four corners. Moreover, the reinforcement was placed with the PGA mesh side down on the abdominal wall (Figures 2(b) and 2(c)). In the PGA alone group, only the PGA mesh was used (Figure 2(a)). The laparotomy wound was closed with 4-0 polyamide sutures in two layers. After the surgery, all rats were bred under the standard SPF conditions.

### 2.5. Evaluations of Adhesion

#### 2.5.1. Macroscopic Evaluations

Adhesion was assessed 2, 4, and 8 weeks after surgery. The rats received isoflurane inhalation anesthesia and were killed humanely by administering a lethal dose of sodium pentobarbital (3.5 mg/kg of body weight) intra-abdominally. Adhesion between each PGA mesh and intra-abdominal organs was scored macroscopically (0–4, according to the extent and severity of adhesion); the scoring system was modified from the Adhesion Score of the Surgical Membrane Study Group (Table 1) [4, 15]. The persons scoring the adhesion were blinded to the rats' group assignment.

#### 2.5.2. Microscopic Analyses

All rats were subjected to the microscopic study. The abdominal wall with PGA mesh fabric was removed en bloc, that is, along with adhering organs and tissues. The specimens were fixed in 10% formalin solution and prepared into thin slices (4 *μ*m thick) stained with hematoxylin eosin. Histological evaluations, including the status of the healing process of the tissues surrounding the materials, were performed in a blinded manner. We quantitatively classified the status of residual alginate, macrophages ingesting alginate, fibroblasts, and collagen fibers by histological scores (Table 2) [4]. To evaluate mesothelial regeneration, immunological staining with antihuman mesothelial cell antibody (HBME-1, Serotec, Japan) was performed. The persons scoring the adhesion were blinded to the rats' group assignment.

### 2.6. Statistical Analyses

Statistical analyses were performed using the software “StatMate®” (ATMS Co., Ltd., Tokyo, Japan). Homoscedasticity of data was confirmed by Bartlett's test before performing analysis of variance (ANOVA). Parametric data were determined by Tukey's test after one-way ANOVA. Nonparametric data were determined by Kruskal-Wallis method followed by two-sided Mann–Whitney *U* tests. Differences were generally considered statistically significant when *p* value was <0.05.

## 3. Results

### 3.1. Macroscopic Evaluations of Adhesion (Table 3)

PGA mesh alone group exhibited severe and wide adhesion to the visceral organs throughout the observation period. Na-alg (*p* < 0.01) limited the extent and severity of adhesion throughout the observation period. Ca-alg significantly (*p* < 0.05) limited the extent of adhesion only at 8 weeks after surgery.

### 3.2. Microscopic Analyses of Adhesion (Table 4)

**Figure 3:**
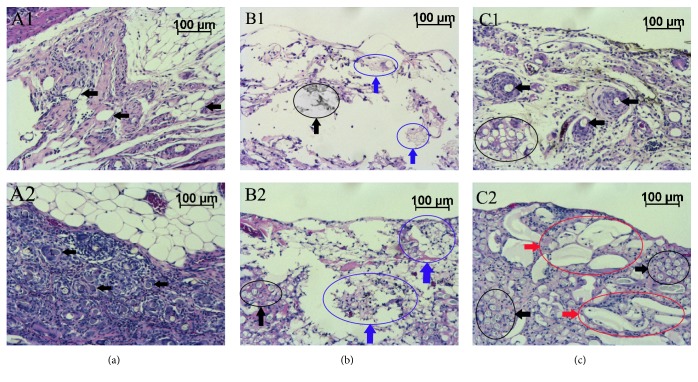
Microscopic findings of the explanted tissues surrounding the materials (H-E staining): (a) PGA mesh at 2 weeks (A1) and 8 weeks (A2). (b) Na-alg at 2 weeks (B1) and 8 weeks (B2). (c) Ca-alg at 2 weeks (C1) and 8 weeks (C2). Black arrows (***←***) show PGA fibers. Blue arrows show macrophages ingesting alginate. Red arrows show alginate surrounded by fibroblasts and collagen fibers.

PGA mesh (Figures 3(A1) and 3(A2)): Almost all PGA fibers remained at the implanted site at 2 to 8 weeks; PGA fibers, which were lost in the process of specimen fixation, were shown as void spaces or purple substances. Abundant fibroblasts and collagen fibers around the PGA mesh fabrics were noted throughout the observation period.

Na-alg (Figures 3(B1) and 3(B2)): Few fibroblasts and collagen fibers inside and around the material 2 weeks after surgery were observed (Figure 3(B1)); however, the fibroblasts and collagen fibers increased sparsely at 8 weeks (Figure 3(B2)). Na-alg remained sparse at the implanted site, and most of the material was ingested by macrophages in 2 weeks.

Ca-alg (Figures 3(C1) and 3(C2)): Fibroblasts around the PGA mesh fabrics gradually increased and abundant collagen fibers surrounding PGA fibers were seen throughout the observation period. Macrophages also ingested the material, similar to the Na-alg group; however, some Ca-alg remained until 4 weeks.

**Figure 4:**
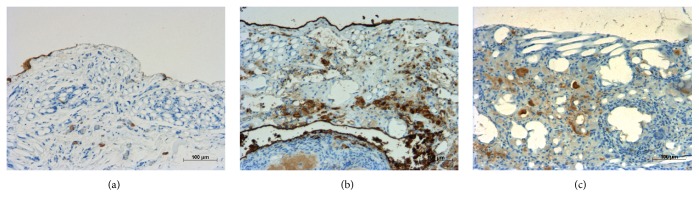
Macroscopic findings of immunostaining (HBME-1) at 8 weeks: (a) PGA mesh, (b) Na-alg, and (c) Ca-algorithm.

The microscopic views of HBME-1 staining at 8 weeks are shown in Figure 4. Matured mesothelium was regenerated at the implanted site in the Na-alg group (Figure 4(b)). In the Ca-alg group, no mesothelial layer was formed after 8 weeks (Figure 4(c)).

## 4. Discussion

PGA mesh fabric is widely used as tissue reinforcement for surgeries. However, PGA-induced adhesion and its complications (ileus, infertility, and abdominal pain) are issues that need to be addressed [5, 6, 16, 17]. PGA-induced adhesion could be attributed to the following factors: (1) inflammation due to xenobiotic reaction and (2) inflammation due to decreased tissue pH around the PGA mesh [6].

Implanted biomaterials serve as matrices for cell adhesion and growth. Macrophages and fibroblasts migrate around the materials by xenobiotic reaction for over 2 weeks. Tissue acidity increases resulting from nonenzymatic degradation of PGA. Decreased tissue pH causes local inflammation, which is followed by fibroblast migration and collagen fiber proliferation around the PGA mesh. We reported the biodegradation behaviors of the PGA mesh (NEOVEIL®) and pH changes of PBS following glycolic acid production. pH level decrease began at 4 weeks and reached a minimum of 6.2 at 7 weeks, and pH increase was noted at 8 weeks (unpublished data).

Considering the properties of PGA and to prevent PGA-induced adhesion, the additional materials should have the ability to provide a physical barrier that has the least adhesion-inducing effects, to prompt regeneration of the peritoneum around the PGA reinforcement, and to degrade the materials as soon as possible after tissue repair. PGA mesh fabrics showed strong adhesion-inducing effects in this study. Thus, preventing adhesion after surgical application of a reinforcement made of PGA is an important concern.

Alginate is known to have antiadhesive effects [3, 18–20]. Because of its biocompatibility, low toxicity, and relatively low cost, it is widely used in medical fields. Alginic acid is a macromolecular polysaccharide and a linear copolymer with homopolymeric blocks of two types of uronic acid: (1-4)-linked *β*-D-mannuronate (M) and its C-5 epimer *α*-L-guluronate (G). Uronic acid is an ion-exchange agent with carboxyl groups between protons and cations, such as Na^+^ or Ca^2+^. Na-alg is soluble and the carboxyl group in alginate is linked to Na^+^. Divalent cations, such as Ca^2+^, bind to the *α*-L-guluronic acid blocks in a highly cooperative manner. Ca-alg is insoluble and serves as a matrix for cell adhesion like fibroblasts and collagen fibers.

Fibroblasts and collagen fibers at the implanted sites were more abundant in the Ca-alg group than in the Na-alg group. It is suggested that Na-alg has high solubility in aqueous solution and diffuses easily in the abdominal cavity and that Ca-alg has lower solubility than Na-alg, although cross-linking density is low [21]. Alginate is absorbed mainly by the lymphatics. According to Supersaxo et al. [22], substances with molecular weight >16,000 are absorbed and drained mainly by the lymphatics. Calcium ion flowing out from Ca-alg in the abdominal cavity should activate prothrombin and accelerate fibrin formation.

Moreover, alginate itself is insoluble; thus, a new device of alginate salt that is easy to use during surgery is needed. In our previous study [4], the ability of alginate salt to prevent PGA-induced adhesion was evaluated. Antiadhesive effects of Ca^2+^ cross-linked alginate gel with good retentivity applied on PGA mesh fabrics were examined. The efficacy in inhibiting PGA-induced adhesion depended on Ca^2+^ cross-linked density. Alginate salt with low Ca^2+^ cross-linked density showed a strong antiadhesive effect, whereas that with high Ca^2+^ cross-linked density had a weak effect. Na-alg powder and solution showed the strongest effects in preventing PGA-induced adhesion. However, Na-alg powder and solution or Ca-alg gel is difficult to use during surgery and has poor retentivity. To solve these problems, we developed a reinforcement composed of PGA mesh and alginate salt and evaluated the adhesion-suppressing effect.

In the PGA mesh alone group, the adhesion formation effect is strong, which is similar to the results of our previous examination. Na-alg showed the strongest antiadhesive effect from the early postoperative period. Ca-alg showed a moderate antiadhesive effect after an 8-week-long observation; however, no significant effect in preventing adhesion in the earlier period was noted. Generally, the effect of Ca-alg foam in preventing adhesion is inferior to that of Ca-alg gel with low Ca^2+^ cross-linked density [4].

Early regeneration of the mesothelium and suppression of local inflammation by PGA were considered to contribute to the antiadhesive effect of Na-alg foam in this study. In the Na-alg group, mesothelial regeneration was already observed in the second week, and matured mesothelial layer developed in 8 weeks. In the PGA alone group, the regeneration of the mesothelium was hardly found. In the Ca-alg group, immature mesothelium formed in 8 weeks. Na-alg provided the appropriate scaffold for tissue regeneration and barrier effects against PGA fibers.

In addition, in the PGA mesh alone group, fibroblasts and collagen fibers proliferated since the early period after surgery and increased further with time. In the Na-alg group, fibroblasts and collagen fibers were scarce (Figure 3). In the Ca-alg group, fibroblasts and collagen fibers proliferated moderately. Doyle et al. reported that fibroblasts proliferate more successfully on Ca-alg than on Na-alg [23]. Suppression of fibroblast and collagen fiber proliferation was considered one of the vital factors for the prevention of PGA-induced adhesion. Fibroblasts and collagen fibers play a major role in adhesion formation and their excessive proliferation results in postoperative adhesion. Thus, inhibiting their increase is the key to preventing PGA-induced adhesion. In the present study, fibroblast and collagen fibers proliferation was evaluated by histological study. Quantitative evaluation of collagen fibers production should bring useful information to analyze the mechanism of the inhibition of PGA-induced adhesion. It would be helpful to evaluate the level of hydroxyproline in the adhesion or in the peritoneal surface to track collagen fiber synthesis. The purpose of this study is to evaluate an inhibiting effect of PGA-induced adhesion with the sodium alginate foam. We could not step deeply into the relations with the antiadhesive effects and collagen-producing restraint, but we should investigate a quantitative evaluation of hydroxyproline level in the injured site in the further study.

Regarding alginate degradation, Na-alg was ingested by macrophages from the early stage of the postoperative period and few residues were noted. Ca-alg ingestion by macrophage was less than Na-alg ingestion, and the subsequent residues were higher in the Ca-alg group. Moreover, the solubility of alginate salts may partly contribute to the amount of alginate residue. To prevent long-term xenobiotic reactions, alginate should be degraded as soon as possible after completion of mesothelial regeneration. Thus, the rapid degradation of Na-alg would be another advantage.

The efficacy of the newly developed reinforcement, PGA mesh unified with Na-alg foam, should be evaluated by using it in surgeries that need reinforcements in humans.

## 5. Conclusions

The reinforcement composed of PGA mesh unified with Na-alg foam strongly inhibited PGA-induced adhesion and showed excellent handling during surgery. Moreover, it can be easily applied with a one-step procedure. Its clinical use is expected in the future.

## Figures and Tables

**Table 1:** Adhesion score.

Category and description score	Score
*Extent*	
No involvement	0
≤25% of the site involved	1
≤50% of the site involved	2
≤75% of the site involved	3
≤100% of the site involved	4
*Severity*	
No adhesion present	0
Adhesion falls apart	1
Adhesion can be lysed with traction	2
Adhesion requiring < 50% sharp dissection	3
Adhesion requiring > 50% sharp dissection	4

**Table 2:** The scores by histological findings.

Category and description	Score
*Residual alginate*	
Sparse residual alginate	±
Focal residual alginate	+
Diffuse residual alginate	++
*Macrophages ingesting alginate*	
No alginate ingestion	−
Sparse macrophages ingesting alginate	±
Focal macrophages ingesting alginate	+
Diffuse macrophages ingesting alginate	++
*Fibroblasts*	
Sparse fibroblasts	−
Focal fibroblasts between PGA fibers	±
Diffuse fibroblasts between PGA fibers	+
Diffuse fibroblasts and focal connective tissue formation with collagen fibers	++
Diffuse fibroblasts and diffuse connective tissue formation with collagen fibers	+++
*Collagen fibers*	
Sparse collagen fibers	−
Focal collagen fibers between PGA fibers	±
Diffuse collagen fibers between PGA fibers	+
Diffuse collagen fibers and diffuse connective tissue formation with fibroblasts	++
Diffuse collagen fibers and focal connective tissue formation with fibroblasts	+++

**(a):** 

Experimental group	Extent of adhesion		
	2 weeks	4 weeks	8 weeks
PGA alone	2.67 ± 1.03	3.67 ± 0.52	4.00 ± 0
Na-alg	0.67 ± 0.52^*∗*^	0.50 ± 0.84^*∗∗*^	0.17 ± 0.41^*∗∗*^
Ca-alg	2.00 ± 1.26	2.50 ± 1.64	1.17 ± 1.83^*∗*^

**(b):** 

Experimental group	Severity of adhesion		
	2 weeks	4 weeks	8 weeks
PGA alone	3.00 ± 0.89	3.00 ± 0.89	3.83 ± 0.41
Na-alg	1.00 ± 0.89^*∗*,††^	0.5 ± 0.84^*∗∗*^	0.17 ± 0.41^*∗∗*^
Ca-alg	3.17 ± 0.41	2.50 ± 1.76	1.17 ± 1.83

Mean ± SD; versus PGA alone: ^*∗*^*p* < 0.05 and ^*∗∗*^*p* < 0.01; versus Na-alg: ^††^*p* < 0.01

**Table 4:** Summary of the scores by histological findings at 2, 4, and 8 weeks after surgery.

Experimental group	Category	2 weeks	4 weeks	8 weeks
Na-alg	Residual alginate	+	+	±
	Macrophages ingesting alginate	++	++	+
	Fibroblasts	−	±	+
	Collagen fibers	−	±	+

Ca-alg	Residual alginate	+++	++	+
	Macrophages ingesting alginate	++	+	±
	Fibroblasts	+	++	+++
	Collagen fibers	++	+++	+++
